# *B*_0_ navigator enables respiratory motion navigation in radial stack‐of‐stars liver Look‐Locker *T*_1_ mapping

**DOI:** 10.1002/mrm.30567

**Published:** 2025-05-20

**Authors:** Jonathan Stelter, Kilian Weiss, Veronika Spieker, Julia A. Schnabel, Rickmer F. Braren, Dimitrios C. Karampinos

**Affiliations:** ^1^ Institute for Diagnostic and Interventional Radiology, School of Medicine and Health, TUM University Hospital Technical University of Munich Munich Germany; ^2^ Philips GmbH Market DACH Hamburg Germany; ^3^ Institute of Machine Learning for Biomedical Imaging Helmholtz Munich Neuherberg Germany; ^4^ School of Computation, Information and Technology Technical University of Munich Munich Germany; ^5^ School of Biomedical Engineering and Imaging Sciences King's College London London UK; ^6^ Munich Institute of Biomedical Engineering Technical University of Munich Garching Germany; ^7^ Munich Data Science Institute Technical University of Munich Garching Germany; ^8^ Present address: Institute for Diagnostic and Interventional Radiology TUM University Hospital Munich Germany

**Keywords:** free‐breathing, gradient echo imaging, self‐navigation, water‐fat separation

## Abstract

**Purpose:**

To develop a $B_{0}$ self‐navigation approach to estimate respiratory motion for motion‐corrected liver $T_{1}$ mapping using a Look‐Locker acquisition with radial stack‐of‐stars trajectory.

**Methods:**

The proposed method derives 1D field‐map profiles from the oversampled k‐space center to estimate a normalized breathing curve and the $B_{0}$ variation amplitude for each slice and coil. $B_{0}$ drift and contrast variations, inherent to the Look‐Locker acquisition, were modeled and corrected by fitting and demodulating drift and offset terms. The breathing curve was employed to bin data into motion states for motion‐resolved reconstruction, followed by water‐specific $T_{1}$ mapping. Simulations with an anatomical body model and in vivo experiments with a Look‐Locker multi‐echo gradient echo sequence were performed to validate the technique. The estimated normalized breathing curve was compared with magnitude‐ and phase‐based self‐navigation approaches using principal component analysis.

**Results:**

The proposed $B_{0}$ self‐navigation reliably estimated the normalized breathing curve and the $B_{0}$ variation amplitude in simulations and in vivo. $B_{0}$ variation amplitudes increased with greater tissue displacement, with median values across slices and coils ranging from 4 to 15 Hz at 3 T in volunteers. Motion‐resolved reconstruction using the estimated breathing curve reduced motion artifacts and improved image and $T_{1}$ mapping quality compared to motion‐averaged reconstruction.

**Conclusion:**

$B_{0}$ self‐navigation allows estimation of respiratory motion in acquisitions with varying contrast and quantifies the $B_{0}$ variation amplitude, providing a possible surrogate signal for tissue displacement and enabling self‐gated liver $T_{1}$ mapping using a Look‐Locker approach.

## 1 INTRODUCTION

Free‐breathing acquisitions are of increasing interest in abdominal imaging, enabling high‐resolution volumetric imaging and quantitative relaxation mapping that may not be feasible in a breath‐hold. Radial stack‐of‐stars trajectories are particularly well‐suited for such applications due to their inherent oversampling of the k‐space center, which also enables self‐navigation of respiratory motion.^1^ However, estimating a reliable motion signal may be challenging in acquisitions with varying contrast.

Look‐Locker‐based $T_{1}$ mapping is an example of such an acquisition with varying contrast across different inversion times ($T_{I}$) and has previously been combined with a stack‐of‐stars trajectory to perform liver water‐specific $T_{1}$ ($wT_{1}$) mapping.^2^, ^3^, ^4^, ^5^ Yet, previous approaches have relied on external motion signals such as bellows,^2^, ^4^ the pilot tone,^5^ or model‐based motion estimation from contrast‐weighted magnitude data,^3^, ^6^ to allow for motion‐resolved reconstruction.

Separately, a phase‐based $B_{0}$ self‐navigation approach leveraging the 1D oversampled k‐space in gradient echo radial stack‐of‐stars acquisitions has been proposed for estimating and correcting temporal $B_{0}$ variations in body regions with minimal tissue displacement and respiratory motion‐induced $B_{0}$ fluctuations such as the neck and shoulder region.^7^ This $B_{0}$ self‐navigation technique has not yet been applied in anatomies such as the liver, where substantial tissue displacement occurs during the respiratory cycle. Additionally, phase‐based approaches may be advantageous in Look‐Locker acquisitions with varying contrast weightings, where magnitude‐based motion estimation may be complicated.

This work aims to develop a $B_{0}$ self‐navigation method for estimating respiratory motion in acquisitions with different contrast weightings and apply the method in free‐breathing whole‐liver $wT_{1}$ mapping.

## 2 THEORY

The $B_{0}$ navigator $f_{B,nav}$ has been recently proposed to estimate and correct temporal $B_{0}$ variations in radial stack‐of‐stars imaging using the 1D oversampled k‐space in the z‐direction.^7^ The $B_{0}$ navigator is estimated from the navigator signal of coil j, which is the average signal $s_{nav,j}(z,T_{E})$ in the radial xy‐plane weighted by the coil sensitivity $c_{j}(x,y,z)$:

(1) $$s_{nav,j}(z,T_{E})=\int c_{j}(x,y,z)s(x,y,z,T_{E})dxdy,$$

at the echo time $T_{E}$. In gradient echo imaging, the signal is modeled as the complex sum of signals $\varrho_{i}(x,y,z)$ from different chemical species with a chemical shift‐induced resonant frequency relative to the center frequency $f_{i}$, and a coefficient $\eta (x,y,z)=i2\pi f_{B}(x,y,z)-R_{2}^{*}(x,y,z)$, which accounts for the apparent transverse relaxation $R_{2}^{*}(x,y,z)$ (assumed common for all chemical species) and field inhomogeneity $f_{B}(x,y,z)$:

(2) $$s(x,y,z,T_{E})=\underset{i}{\sum }\varrho_{i}(x,y,z)e^{i2\pi f_{i}T_{E}}e^{\eta (x,y,z)T_{E}}.$$

Typically, the signal is modeled for two species, water $\varrho_{W}$ and fat $\varrho_{F}$, with a multi‐peak fat spectrum $d(T_{E})=\sum_{p=1}^{P}\alpha_{p}e^{i2\pi f_{p}T_{E}}$ and $\sum_{p=1}^{P}\alpha_{p}=1$.^8^, ^9^

To understand the main factors affecting the $B_{0}$ navigator signal in the radial stack‐of‐stars acquisition, we first study 2D examples. Specifically, we use a 2D object and determine the equivalent navigator signal, corresponding to the $B_{0}$ value extracted based on the multi‐echo signal at the k‐space center.

### 2.1 $B_{0}$ navigator in simplified two‐species model

**FIGURE 1:**
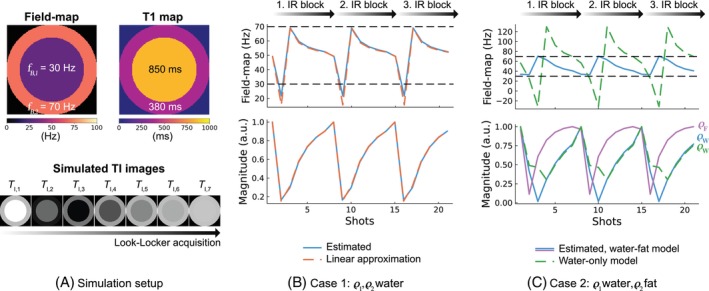
Effect of contrast variations on the $B_{0}$ navigator in a 2D object. The $B_{0}$ navigator was estimated similarly to the other experiments using a graph‐cut algorithm with a water‐only or a water‐fat signal model. (A) Simulated Look‐Locker signal for two tissues ($\varrho_{1}$, $\varrho_{2}$) with different spatial distribution, $B_{0}$ and $T_{1}$ values. (B) Both tissues are modeled as water‐based, with no additional chemical shift. The estimated $B_{0}$ navigator (${\tilde{f}}_{B,nav}$) and the signal magnitude variations are shown over the acquired shots, with contrast changes occurring over 7 shots. The approximation from Equations 4 and 5 is included as a reference. (C) $\varrho_{2}$ is modeled as fat with a multi‐peak fat spectrum, resulting in different ${\tilde{f}}_{B,nav}$ variations compared to the first case. As a comparison, the estimated Dixon‐based $B_{0}$ navigator is compared to a water‐only signal model used in the estimation of the $B_{0}$ navigator, resulting in stronger estimated ${\tilde{f}}_{B,nav}$ variations.

Let us consider a 2D object ($s_{nav}(z,T_{E})=s_{nav}(T_{E})$ Figure 1A,B) consisting of two water species ($\varrho_{1}$ and $\varrho_{2}$) with different spatial distributions, 2D areas ($A_{1}$ and $A_{2}$) and field‐maps ($f_{B,1}$ and $f_{B,2}$). Neglecting $T_{2}^{*}$ decay and assuming a single coil with uniform sensitivity (c(x,y,z)=1), the navigator signal for a proton density‐weighted acquisition becomes:

(3) $$\begin{array}{ll} s_{nav}(T_{E}) & =\int_{A_{1}}\varrho_{1}e^{i2\pi f_{B,1}T_{E}}dxdy+\int_{A_{2}}\varrho_{2}e^{i2\pi f_{B,2}T_{E}}dxdy\\ & =\varrho_{1}A_{1}e^{i2\pi f_{B,1}T_{E}}+\varrho_{2}A_{2}e^{i2\pi f_{B,2}T_{E}}, \end{array}$$

For small field‐map‐induced phase terms ($f_{B,1}T_{E}<<1$ and $f_{B,2}T_{E}<<1$), the navigator signal can be approximated as single‐species signal using a first‐order Taylor expansion (see also Supporting Information):

(4) $$s_{nav}(T_{E})\approx (\rho_{1}A_{1}+\rho_{2}A_{2})e^{i2\pi f_{B,nav}T_{E}}.$$

The average field and $B_{0}$ navigator, $f_{B,nav}$, is a weighted average of the individual field‐maps:

(5) $$\begin{array}{ll} f_{B,nav}(\{\varrho_{i}\},\{A_{i}\},\{f_{B,i}\}) & =\frac{\varrho_{1}A_{1}}{\varrho_{1}A_{1}+\varrho_{2}A_{2}}f_{B,1}\\ & \;+\frac{\varrho_{2}A_{2}}{\varrho_{1}A_{1}+\varrho_{2}A_{2}}f_{B,2}. \end{array}$$

The $B_{0}$ navigator is computed based on the integration of the complex signal and is therefore dependent on the underlying composition of the two‐species model.

### 2.2 $B_{0}$ navigator in water‐fat model

When water and fat species are considered (Figure 1C), Equation (5) is modified to account for the multi‐peak fat spectrum and echo times, resulting in the following dependencies: $f_{B,nav}(\{\varrho_{i}\},\{A_{i}\},\{f_{B,i}\},d(T_{E}))$.

The $B_{0}$ navigator is again computed based on the integration of the complex signal and is therefore dependent on the underlying composition of the water‐fat model, in particular the fat spectrum $d(T_{E})$ with the employed echo times $T_{E}$.

### 2.3 $B_{0}$ navigator in scans with varying contrast

For scans with varying contrast, the signal $\varrho_{i}$ for species i may vary during the scan, for example, with the inversion time $T_{I}$ in Look‐Locker experiments. This leads to a dependence of the $B_{0}$ navigator on the inversion time: $f_{B,nav}(\{\varrho_{i}(T_{I})\},\{A_{i}\},\{f_{B,i}\},d(T_{E}))$.

The average field for each coil and slice can be estimated as a function of time t from the oversampled 1D k‐space acquired in each shot.^7^ This field‐map can be decomposed into a contrast‐dependent offset $\alpha (T_{I})$ and the temporal $B_{0}$ variations $\Delta f_{B}(t)$:

(6) $$f_{B,nav}(t)=\alpha (T_{I})+\Delta f_{B}(t),$$

with the temporal $B_{0}$ variations assumed to be spatially approximately homogeneous for each slice and sensitivity of the receive coil.

The contrast‐dependent offset $\alpha (T_{I})$ is constant over a given inversion time $T_{I}$ and captures the effect of the varying contrast on the $B_{0}$ navigator (the $B_{0}$ navigator is based on the integration of complex signal along the x and y axes as shown before). The temporal $B_{0}$ variations capture estimated variations independent of the varying contrast, such as a drift induced by gradient heating or breathing‐induced $B_{0}$ variation. The linear drift is modeled with slope β and the breathing‐induced $B_{0}$ variation is modeled with the breathing curve b(t) and the $B_{0}$ variation amplitude γ:

(7) $$\Delta f_{B}(t)=\beta t+\gamma b(t)$$

The $B_{0}$ navigator is therefore presently modeled as follows for each slice and coil:

(8) $$f_{B,nav}(t)=\alpha (T_{I})+\beta t+\gamma b(t),$$

To ensure separation of the different components, b(t) is normalized to an interval [−1,1] and assumed to be centered around zero and independent of the contrast offset and linear drift. The proposed fitting procedure is initially performed with the breathing curve b(t) set to zero, after which b(t) and its $B_{0}$ variation amplitude γ are estimated using an iterative scheme.

## 3 METHODS

### 3.1 $B_{0}$ self‐navigation and respiratory motion estimation

The oversampled k‐space along the $k_{z}$‐direction was extracted from the center of each spoke, and 1D magnitude and phase projections were computed using the inverse Fourier transform. First, low‐frequency components that vary with the angular increment of the spokes were removed using an orthogonal projection.^7^, ^10^ Coil‐wise water‐fat separation was performed using a single‐resolution variable‐layer single‐min‐cut graph‐cut algorithm,^11^ with smoothness enforced along the z‐direction and the temporal shot dimension.^7^ The estimated field map per coil, varying with the slice location and time, constitutes the $B_{0}$ navigator signal to be analyzed further.

The model in Equation (8) is therefore extended with the $B_{0}$ navigator per coil j as a function of both slice location z and time t:

(9) $$f_{B,nav,j}(t,z)=\alpha_{j}(T_{I},z)+\beta_{j}(z)t+\gamma_{j}(z)b(t),$$

**FIGURE 2:**
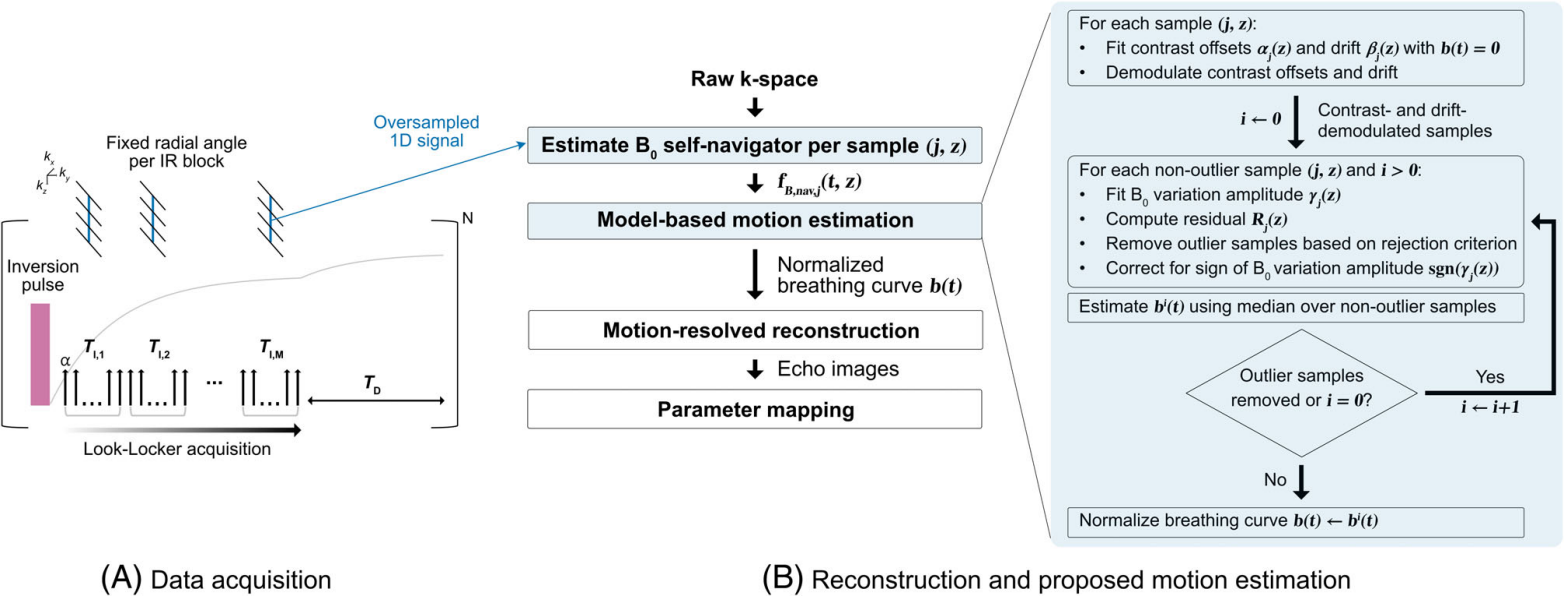
(A) Data acquisition scheme based on an inversion recovery Look‐Locker sequence. (B) Proposed $B_{0}$ self‐navigation method and image reconstruction. The $B_{0}$ navigator was estimated from the oversampled k‐space of the radial stack‐of‐stars trajectory for each coil j and slice location z using a graph‐cut algorithm. The normalized breathing curve b(t) was estimated from the $B_{0}$ navigator by fitting contrast offsets $\alpha_{j}(T_{I},z)$ and a linear $B_{0}$ drift coefficient $\beta_{j}(z)$. These components were demodulated from the sample, and the $B_{0}$ variation amplitude $\gamma_{j}(z)$ was estimated. Samples with large residuals were excluded iteratively to refine the estimate of b(t), which is the median of all demodulated samples. The resulting motion curve was employed to bin k‐space data into motion states. Finally, a motion‐resolved reconstruction and water‐specific $T_{1}$ mapping were performed.

A single breathing curve b(t) was estimated from the 1D field‐map profiles, assuming the model in Equation (9) (Figure 2B). The estimation process involved two main steps:

First, contrast offsets $\alpha_{j}(T_{I},z)$ and the $B_{0}$ drift coefficients $\beta_{j}(z)$ were estimated using a least squares fit with b(t) set to zero. The resulting residual represents the navigator signal with the contrast offset and linear drift demodulated. An initial estimate for b(t) was obtained by computing the median of the demodulated samples from all slices and coils.

Second, an iterative scheme was employed to refine the initial estimate for b(t). In each iteration, the $B_{0}$ variation amplitude $\gamma_{j}(z)$ was estimated via least squares. Outlier samples with large residuals $R_{j}(z)$ were excluded based on a rejection criterion:

(10) $$R_{j}(z)>Q_{3}+1.5(Q_{3}-Q_{1})$$

with $Q_{1}$ and $Q_{3}$ the lower and upper quartile of the set $\left\{R_{j}(z)|(j,z)\in U\right\}$ with U the set of all accepted samples. The residual $R_{j}(z)$ was computed based on the mean squared error between the estimated field‐maps ${\tilde{f}}_{B,nav,j}(t_{i},z)$ and the fitted model ${\hat{f}}_{B,nav,j}(t_{i},z)$:

(11) $$R_{j}(z)=w_{j}(z)\overset{N*M}{\underset{i}{\sum }}{\left({\tilde{f}}_{B,nav,j}(t_{i},z)-{\hat{f}}_{B,nav,j}(t_{i},z)\right)}^{2},$$

with the weights $w_{j}(z)=\frac{1}{N*M}$ for $|\gamma_{j}(z)|<1$ and $w_{j}(z)=\frac{1}{|\gamma_{j}(z)|*N*M}$ for $|\gamma_{j}(z)|>1$, the number of inversion‐recovery blocks N and the number of inversion contrasts per block M. N∗M denotes the total number of shots in the Look‐Locker acquisition.

During each iteration, the sign of the fitted $\gamma_{j}(z)$ was used to align the signs of the demodulated samples. Subsequently, the estimate for b(t) was refined by recomputing the median over the remaining sign‐corrected demodulated samples. The iterative scheme was terminated when no samples were rejected, and the resulting breathing curve was normalized based on the 5th and 95th percentiles to a [−1,1] interval.

### 3.2 Free‐breathing Look‐Locker acquisition

A Look‐Locker scheme was combined with a bipolar multi‐echo gradient echo acquisition using a radial stack‐of‐stars trajectory (Figure 2A). Seven different $T_{I}$ contrasts were acquired following an inversion preparation pulse (M=7), with a shot duration of $T_{\text{shot}}=297\;\text{ms}$ per $T_{I}$. Each inversion‐recovery block consisted of $T_{\text{block}}=T_{I,1}+MT_{\text{shot}}+T_{D}=3.5\;s$, where $T_{I,1}$ represents the delay between inversion pulse and the first shot and $T_{D}$ represents the free recovery delay.

During each shot, profiles along the $k_{z}$‐direction were acquired at a fixed radial spoke angle with centric profile ordering. The spoke angle was varied across blocks using pseudo‐golden‐angle ordering.^12^ The acquisition was performed during free breathing, without respiratory triggering.

### 3.3 Reconstruction pipeline

The $B_{0}$ navigator and the normalized breathing curve b(t) were estimated from raw k‐space data (Section 3.1). Four motion states were defined using k‐medoids clustering^13^ based on the estimated normalized breathing curve.

Image reconstruction was performed using an ADMM optimizer solving the following problem:

(12) $$\begin{array}{ll} x & ={\arg\;\min}_{x^{\prime }}||ℱ𝒮x^{\prime }-y|{|}_{2}^{2}+ℛ(x^{\prime }) \end{array}$$

(13) $$\begin{array}{ll} ℛ(x) & =\lambda_{1}||{\text{TV}}_{\text{3D}}(x)|{|}_{1}+\lambda_{2}||{\text{TV}}_{\text{1D}}(x)|{|}_{1} \end{array}$$

with x the complex reconstructed echo images, y the multi‐coil k‐space, ℱ the inverse Fourier transform along $k_{z}$ and the non‐uniform fast Fourier transform (NUFFT) per slice, and 𝒮 the coil sensitivity maps, estimated from a pre‐scan.

Two reconstructions were performed: First, a motion‐averaged reconstruction with all data combined into a single motion state and 3D TV regularization applied in image space ($\lambda_{1}=0.5$, $\lambda_{2}=0$), and, second, a $B_{0}$‐navigated reconstruction with data separated into four motion states and 1D TV regularization applied in the respiratory dimension in addition to the spatial 3D TV regularization ($\lambda_{1}=0.5$, $\lambda_{2}=1.0$).

Water‐fat separation of the reconstructed images was performed using a multi‐resolution graph‐cut algorithm^14^ and $T_{1}$ fitting was conducted using a model that accounts for incomplete $T_{1}$ recovery.^15^ A 9‐peak fat model was assumed throughout this study.^16^, ^17^

The source code and example data are publicly available: https://github.com/BMRRgroup/B0nav‐LL.

### 3.4 Experiments

#### 3.4.1 Motion simulation

Simulations were performed on an anatomical body model (XCAT phantom^18^) with tissue‐specific proton density, fat fraction, $T_{1}$, $T_{2}^{*}$ and magnetic susceptibility χ values for the abdomen. Respiratory motion was simulated with 10 different motion frames over a respiratory cycle of 5 s, with maximum diaphragm displacement of 15/25 mm and anterior‐posterior expansions of 4/6 mm.

In an additional simulation with maximum diaphragm displacement of 15 mm and an anterior‐posterior expansion of 4 mm, the respiration period was varied to 3.6 s to investigate the effects of a respiratory cycle almost synchronized with the data acquisition. The simulated scan time was varied from the nominal scan time of 7:37 min to 1 min to further investigate the robustness of the estimate for shorter scans.

Magnitude and phase images for different $T_{I}$ contrast weightings and echoes were generated using a water‐fat signal model (Equation (2)) with the water and fat signals simulated based on an inversion recovery signal model. Field inhomogeneities were derived from tissue magnetic susceptibility values χ convoluted with a dipole kernel. k‐space data was forward simulated using a NUFFT with the same trajectory as for the in vivo measurements, and a motion‐combined k‐space dataset was generated based on the sequence timing.

#### 3.4.2 Measurements and comparison methods

Measurements were performed at 3 T (Ingenia Elition X, Philips Healthcare) on five volunteers (number of echoes $N_{\text{TE}}=4$, ${\text{TE}}_{1}/\Delta \text{TE}=1.08/1.07\;\text{ms}$, $\text{FA}=3^{\circ }$, $\text{FOV}=350\times 350\times 200\;{\text{mm}}^{3}$, $3\times 3\times 5\;{\text{mm}}^{3}$ voxel size). The study was approved by the local institutional review board (Klinikum rechts der Isar, Technical University of Munich), and informed consent was obtained from the volunteers. The normalized breathing curve b(t) estimated by the $B_{0}$ navigator was compared to a respiratory motion‐tracking camera (VitalEye, Philips Healthcare) and two different principal component analysis (PCA)‐based self‐navigation methods.

PCA was applied separately to the magnitude (magnitude‐based PCA) or phase (phase‐based PCA) of the oversampled k‐space after an inverse 1D Fourier transform. Before PCA, system imperfections were corrected using an orthogonal projection similar to the $B_{0}$ navigator (Section 3.1), and min‐max normalization and centering around zero was performed for each $T_{I}$ contrast, coil, and slice separately.^1^, ^19^

## 4 RESULTS

### 4.1 Simulation results

**FIGURE 3:**
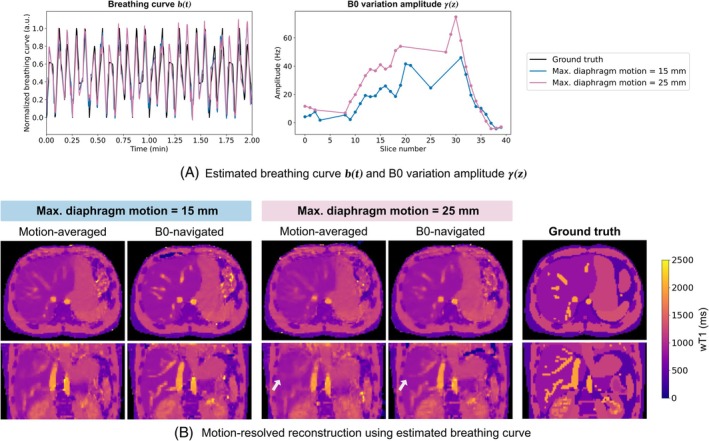
Simulation results using an anatomical body model with respiratory motion at different maximum diaphragm displacements (15 and 25 mm). (A) The estimated normalized breathing curves using the proposed $B_{0}$ navigation method are compared with the ground truth. The $B_{0}$ variation amplitude γ(z) differs between simulations, with larger amplitudes observed for greater tissue displacements. (B) Reconstruction results demonstrate improved agreement with the ground truth for the $B_{0}$‐navigated reconstruction compared to the more blurry motion‐averaged reconstruction.

Figure 3 presents simulation results in an anatomical body model for two amplitudes of respiratory motion. The estimated normalized breathing curves using the proposed $B_{0}$ navigation method closely match the ground truth for both motion scenarios. The proposed method effectively estimated the $B_{0}$ variation amplitude across different slices with median amplitudes of 16 and 26 Hz for maximum diaphragm displacement of 15 and 25 mm, respectively. The $B_{0}$ variation amplitude was not estimated for some slices due to the rejection criterion (Equation (10)).

Reconstructed $wT_{1}$ maps show more pronounced motion‐induced blurring in the motion‐averaged reconstructions for stronger motion (Figure 3B). In contrast, the $B_{0}$‐navigated motion‐resolved reconstruction considerably improved image quality, aligning better with the ground truth. Slight residual blurring remained, especially for the model with larger motion amplitudes.

The effect of a shorter respiratory period of 3.6 s was examined in Supporting Information Figure S1. The estimate of the normalized breathing curve agrees with the ground truth estimate for the long (7:37 min) and the short (1 min) scan, although the respiration period is almost synchronized with the data acquisition (3.5 s interval between two inversion pulses). In addition, the estimated $B_{0}$ variation amplitude is similar to the estimated $B_{0}$ variation amplitude for the longer respiration period of 5 s and shows only minor deviations for the shorter scan. The image quality of the reconstructed $wT_{1}$ maps is comparable for both simulated respiration periods.

### 4.2 In vivo results

**FIGURE 4:**
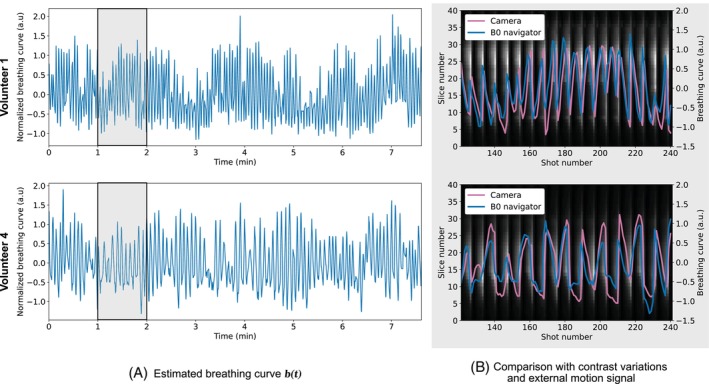
In vivo motion estimation for volunteers 1 and 4. (A) Estimated normalized breathing curve b(t) for the entire scan duration. (B) The slice‐wise magnitude variations from the estimated water navigation signal are shown in a zoomed‐in view. The normalized breathing curve and the external motion‐tracking camera signal are overlayed in blue and pink, respectively.

**FIGURE 5:**
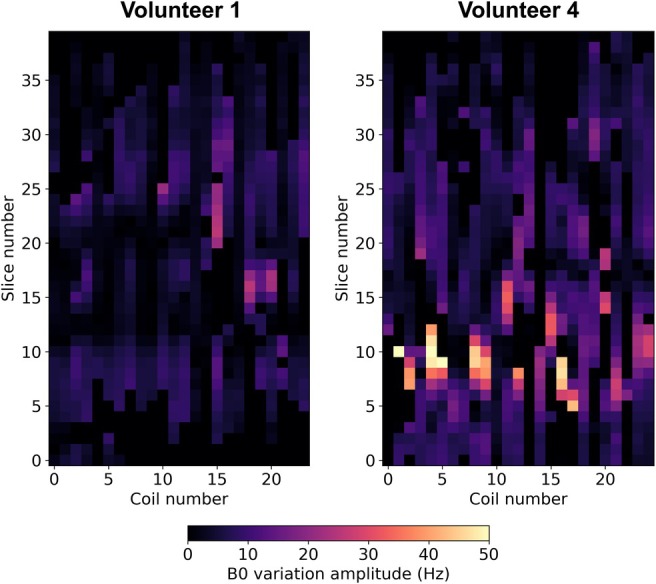
Estimated $B_{0}$ variation amplitude $\gamma_{j}(z)$ per coil j and slice location z. Results are shown for volunteers 1 and 4. Overall, volunteer 4 exhibits larger $B_{0}$ variation amplitudes compared to volunteer 1.

The normalized breathing curves are presented in Figure 4, and the estimated $B_{0}$ variation amplitudes are presented in Figure 5 for two volunteers. The in vivo motion estimation for the additional volunteers is shown in Supporting Information Figure S2. The estimated breathing curves exhibit variability in frequency, amplitude, and periodicity between volunteers. Median $B_{0}$ variation amplitudes across slices and coils range from 4 to 15 Hz, with the lowest values observed in volunteer 1 and the highest in volunteer 2. The comparison with the external motion‐tracking camera (Figure 4B) demonstrates good correspondence, albeit with a potential short delay between both methods. In addition, the grayscale heatmap in subfigure Figure 4B shows magnitude variations in the estimated water navigation signal, revealing a regular pattern every seven shots, consistent with the Look‐Locker acquisition scheme. For volunteer 1, the frequency of the Look‐Locker‐related magnitude variations is similar to the frequency of the normalized breathing curve.

**FIGURE 6:**
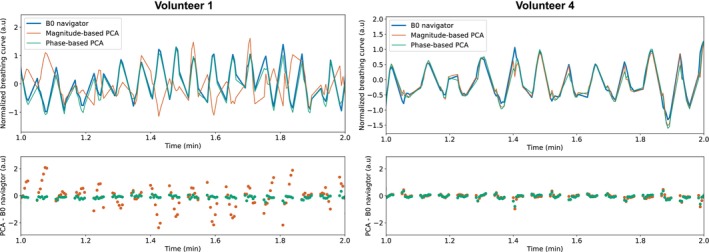
Comparison of the normalized breathing curve b(t) estimated from the $B_{0}$ navigator and two PCA‐based motion estimation approaches. Magnitude‐ and phase‐based PCA approaches use similar pre‐processing with normalization for each $T_{I}$ contrast. The top row shows the estimated breathing curves over a 1‐min interval for volunteers 1 and 4. The bottom row shows the difference between the PCA‐based estimates and the $B_{0}$ navigator. Gaps in the breathing curve sampling correspond to the free recovery delay $T_{D}$ in the pulse sequence scheme.

The estimated normalized breathing curves are compared with magnitude‐ and phase‐based PCA methods in Figure 6 for volunteers 1 and 4, and in Supporting Information Figure S3 for the additional volunteers. The phase‐based approach visually agrees well with the normalized breathing curve from the $B_{0}$ navigator but shows small deviations in waveform, especially for volunteer 3. The magnitude‐based approach shows large deviations for volunteer 1 with a rather irregular waveform.

**FIGURE 7:**
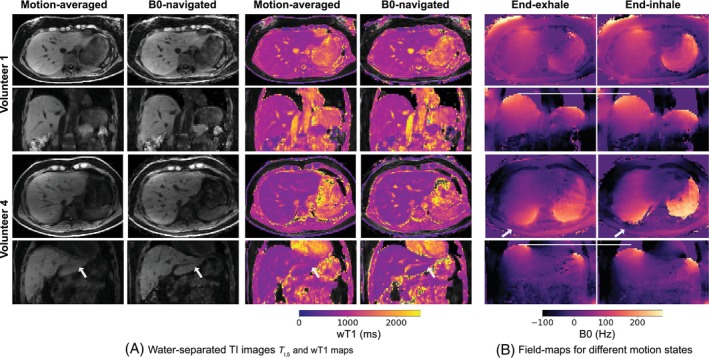
In vivo reconstruction results for volunteers 1 and 4. (A) Motion‐resolved water‐separated $T_{I,5}$ images and $wT_{1}$ maps using the $B_{0}$ navigator are compared to motion‐averaged reconstructions, showing improved image quality (white arrows). (B) Field‐maps for end‐exhalation and end‐inhalation motion states demonstrate minor temporal $B_{0}$ variations for volunteer 1 (median $B_{0}$ variation amplitude γ across slices/coils: 4 Hz) and stronger $B_{0}$ differences along with tissue displacement for volunteer 4 (median $B_{0}$ variation amplitude γ across slices/coils: 7 Hz).

Figure 7A shows reconstructed water‐separated $T_{I}$ images at inversion time $T_{\text{I,5}}=1198\;\text{ms}$ and $wT_{1}$ maps for volunteers 1 and 4 using motion‐averaged and $B_{0}$‐navigated motion‐resolved reconstructions. The $B_{0}$‐navigated reconstructions exhibit enhanced image quality, with improved vessel delineation and reduced blurring, particularly for volunteer 4. A comparison with a motion‐resolved reconstruction using the camera signal is provided in Supporting Information Figure S5. Supporting Information Figure S4 compares both reconstructions for the water‐separated $T_{I}$ images at each inversion time. Field‐maps for end‐exhalation and end‐inhalation motion states are provided in Figure 7B, showing minor differences for volunteer 1 between motion states, while volunteer 4 shows pronounced $B_{0}$ variations between motion states, in addition to observable tissue displacement. The $B_{0}$ navigator estimated a median peak‐to‐peak $B_{0}$ variation amplitude (2γ) across slices and coils of 14 Hz for volunteer 4. The maximal estimated peak‐to‐peak $B_{0}$ variation amplitude across slices and coils for this volunteer was 106 Hz.

## 5 DISCUSSION

A novel $B_{0}$ self‐navigation approach was developed to estimate respiratory motion for liver $T_{1}$ mapping using the oversampled k‐space of the radial stack‐of‐stars trajectory.

The $B_{0}$ self‐navigation technique was initially proposed in radial stack‐of‐stars acquisitions of the neck and shoulder region, where $B_{0}$ variations of up to 25 Hz at 3 T were observed near the lungs, despite minimal tissue displacement in that region.^7^ In contrast, free‐breathing liver imaging involves significant tissue displacement due to respiratory motion. Previous studies have reported $B_{0}$ variations with peak‐to‐peak amplitudes of approximately 20–30 Hz at 3 T in the liver.^20^, ^21^ This work extends the application of the $B_{0}$ navigator to the liver, where breathing‐induced tissue displacement and $B_{0}$ variations are highly correlated.

The proposed $B_{0}$ navigator robustly estimated normalized breathing curves and $B_{0}$ variation amplitudes in simulation and in vivo studies. The application of the $B_{0}$ navigator approach to a Look‐Locker $T_{1}$ mapping sequence introduces additional complexity due to contrast variations in each inversion‐recovery block. The contrast variations primarily influence the magnitude signal, but the estimated $B_{0}$ navigator is also affected due to the fact that the $B_{0}$ navigator is estimated based on the projection of the multi‐echo signal on the z‐axis (see also Section 2).

A model (Equation (9)) was assumed to separate the effects of $B_{0}$ drift, respiratory‐induced $B_{0}$ variations and contrast offsets, which are influenced by the spatially averaged field‐map and depend on species signals and $T_{I}$ values. Unlike prior implementations of the $B_{0}$ navigator,^7^ this work incorporates explicit modeling for temporal $B_{0}$ changes. However, the shape of the breathing curve is not parameterized due to the variability and irregularity of breathing patterns, as also shown in the volunteer study. Further, tissue displacement is not explicitly modeled, and the observed maximum $B_{0}$ variation amplitude may additionally be influenced by tissue displacement.

The parameter estimation followed a two‐step approach. First, the contrast offsets and the $B_{0}$ drift were estimated while setting the breathing curve to zero. Second, the breathing curve was estimated using an iterative scheme based on the residuals of the first step. However, if the respiratory cycle is perfectly periodic and synchronized with the data acquisition, the estimation of a breathing curve becomes infeasible. In practice, respiratory motion is rarely perfectly periodic over extended acquisitions, leading to data sampling of each contrast across different motion states. The robustness of the method in the presence of a respiratory cycle almost synchronized with the data acquisition was demonstrated in a simulation.

The estimation of the $B_{0}$ variation amplitude (parameter γ in Equation (9)) is a notable advantage of this method, since the $B_{0}$ variation amplitude correlates with the maximum tissue displacement in simulations with an anatomical body model and in in vivo experiments. However, future studies may be required, as the maximum measured $B_{0}$ variation amplitude also depends on the coil sensitivities and, therefore, on the coil placement. The estimated $B_{0}$ variation amplitude may enable patient‐specific motion correction methods in future studies,^22^ while the commonly used PCA‐based self‐navigation methods^1^ can only estimate the relative tissue displacement. Similarly, motion‐tracking devices such as optical or pressure‐based sensors can usually only estimate relative motion signals without the ability to compare amplitudes between subjects. The respiratory motion‐tracking camera in this study tracks motion based on the subject's surface^23^ with the advantage that the sensor does not depend on a correct placement in difference to local pressure‐based sensors, such as respiratory belts.^24^ In clinical practice, the motion signals from optical motion‐tracking devices are primarily used qualitatively, for example, for respiratory triggering. Further studies are needed to assess their quantitative potential, for example, for motion‐navigated reconstruction. Differences in waveform or delays compared to self‐navigation techniques are possible, as self‐navigation techniques estimate the motion signal directly from the MR signal without possible delays.

The comparison with PCA‐based self‐navigation showed that phase‐based self‐navigation approaches may be better suited to extract respiratory motion information from scans with varying contrast. The proposed $B_{0}$ navigator is also based on the phase of the oversampled k‐space data, but additionally provides the $B_{0}$ variation amplitude. Furthermore, PCA requires a suitable normalization of the projections for each contrast, similar to the fitting of contrast offsets in the $B_{0}$ navigator approach. Remaining differences in the waveform between PCA‐based methods and the $B_{0}$ navigator may be due to the contrast variations throughout the scan, although normalization was performed. PCA‐based methods can be applied to single‐echo acquisitions, while the proposed $B_{0}$ navigator requires at least three echoes to solve the assumed water‐fat signal model.

The present work has some limitations. First, this study focused on liver $T_{1}$ mapping using a Look‐Locker acquisition. While the proposed method addresses challenges posed by contrast variations, it may not generalize to acquisitions with continuously changing contrast, such as dynamic contrast‐enhanced MRI. Second, regularization in spatial and respiratory dimensions was applied in the motion‐resolved reconstruction without additional regularization along the different $T_{I}$ images due to GPU memory limitations, which may limit the possible maximum undersampling and performance of the image reconstruction. Third, the $B_{0}$ navigator model (Equation (9)) was occasionally unsuitable for certain slices or coils, resulting in outlier rejection due to large residuals. This may be due to locally low coil sensitivity values, large tissue displacement, or the assumption of spatially homogeneous temporal $B_{0}$ variations within each slice and coil. Nevertheless, the estimation of the normalized breathing curve was possible in all experiments with the in vivo estimation of the $B_{0}$ variation amplitude in all slices for at least a subset of coils.

## 6 CONCLUSION

The $B_{0}$ self‐navigation approach from a radial stack‐of‐stars acquisition, originally introduced for the neck and shoulder region with limited tissue displacement, was shown to be robust even in the presence of significant tissue displacement in free‐breathing liver imaging. The proposed method, relying on the $B_{0}$ navigator, enables the estimation of respiratory motion in liver $T_{1}$ mapping with strong contrast variations during the scan, posing an alternative to magnitude‐based motion estimation methods. Moreover, the method directly estimates the amplitude of the breathing‐induced $B_{0}$ variation, which may correlate with absolute tissue displacement.

## CONFLICT OF INTEREST STATEMENT

Kilian Weiss is an employee of Philips GmbH Market DACH, and Dimitrios Karampinos receives grant support from Philips Healthcare.

## FUNDING INFORMATION

This work was supported by the TUM International Graduate School of Science and Engineering, the German Research Foundation and the Philips Healthcare.

## Supporting information


**Data S1.** Supporting Information.

## Data Availability

The source code and example data are publicly available: https://github.com/BMRRgroup/B0nav‐LL (Version v1.0).

## References

[1] Feng L , Axel L , Chandarana H , Block KT , Sodickson DK , Otazo R . XD‐GRASP: Golden‐angle radial MRI with reconstruction of extra motion‐state dimensions using compressed sensing. Magn Reson Med. 2015;75:775‐788. 25809847 10.1002/mrm.25665 PMC4583338

[2] Feng L , Liu F , Soultanidis G , et al. Magnetization‐prepared GRASP MRI for rapid 3D T1 mapping and fat/water‐separated T1 mapping. Magn Reson Med. 2021;86:97‐114. 33580909 10.1002/mrm.28679 PMC8197608

[3] Wang N , Cao T , Han F , et al. Free‐breathing multitasking multi‐echo MRI for whole‐liver water‐specific T1, proton density fat fraction, and R2* quantification. Magn Reson Med. 2021;87:120‐137. 34418152 10.1002/mrm.28970 PMC8616772

[4] Muslu Y , Tamada D , Roberts NT , et al. Free‐breathing, fat‐corrected T1 mapping of the liver with stack‐of‐stars MRI, and joint estimation of T1, PDFF, R2*, and B1+. Magn Reson Med. 2024;92:1913‐1932. 38923009 10.1002/mrm.30182 PMC12207996

[5] Goerke U , Shih SF , Ahanonu E , et al. Free‐breathing stack‐of‐stars look‐locker T1‐mapping with whole liver coverage. Proc Intl Soc Mag Reson Med. 2024;32:0427.

[6] Chen J , Christodoulou AG , Han P , et al. Abdominal MR Multitasking for radiotherapy treatment planning: A motion‐resolved and multicontrast 3D imaging approach. Magn Reson Med. 2024;93:108‐120. 39171431 10.1002/mrm.30256 PMC11518652

[7] Stelter J , Weiss K , Wu M , et al. Dixon‐based B0 self‐navigation in radial stack‐of‐stars multi‐echo gradient echo imaging. Magn Reson Med. 2024;93:80‐95. 39155406 10.1002/mrm.30261

[8] Yu H , Shimakawa A , CA MK , Brodsky E , Brittain JH , Reeder SB . Multiecho water‐fat separation and simultaneous $R_{2}^{*}$ estimation with multifrequency fat spectrum modeling. Magn Reson Med. 2008;60:1122‐1134. 18956464 10.1002/mrm.21737 PMC3070175

[9] Bydder M , Yokoo T , Hamilton G , et al. Relaxation effects in the quantification of fat using gradient echo imaging. Magn Reson Imaging. 2008;26:347‐359. 18093781 10.1016/j.mri.2007.08.012 PMC2386876

[10] Rosenzweig S , Scholand N , Holme HCM , Uecker M . Cardiac and respiratory self‐gating in radial MRI using an adapted singular spectrum analysis (SSA‐FARY). IEEE Trans Med Imaging. 2020;39:3029‐3041. 32275585 10.1109/TMI.2020.2985994

[11] Boehm C , Diefenbach MN , Makowski MR , Karampinos DC . Improved body quantitative susceptibility mapping by using a variable‐layer single‐min‐cut graph‐cut for field‐mapping. Magn Reson Med. 2020;85:1697‐1712. 33151604 10.1002/mrm.28515

[12] Hedderich D , Weiss K , Spiro J , et al. Clinical evaluation of free‐breathing contrast‐enhanced T1w MRI of the liver using pseudo golden angle radial K‐space sampling. Röfo. 2018;190:601‐609. 29534252 10.1055/s-0044-101263

[13] Schubert E , Rousseeuw PJ . Faster k‐Medoids Clustering: Improving the PAM, CLARA, and CLARANS Algorithms. Springer International Publishing; 2019:171‐187.

[14] Stelter JK , Boehm C , Ruschke S , et al. Hierarchical multi‐resolution graph‐cuts for water‐fat‐silicone separation in breast MRI. IEEE Trans Med Imaging. 2022;41:3253‐3265. 35657831 10.1109/TMI.2022.3180302

[15] Wang X , Rosenzweig S , Roeloffs V , et al. Free‐breathing myocardial T1 mapping using inversion‐recovery radial FLASH and motion‐resolved model‐based reconstruction. Magn Reson Med. 2022;89:1368‐1384. 36404631 10.1002/mrm.29521 PMC9892313

[16] Ren J , Dimitrov I , Sherry AD , Malloy CR . Composition of adipose tissue and marrow fat in humans by 1H NMR at 7 Tesla. J Lipid Res. 2008;49:2055‐2062. 18509197 10.1194/jlr.D800010-JLR200 PMC2515528

[17] Hamilton G , Schlein AN , Middleton MS , et al. In vivo triglyceride composition of abdominal adipose tissue measured by 1H MRS at 3T. J Magn Reson Imaging. 2017;45:1455‐1463. 27571403 10.1002/jmri.25453 PMC5332519

[18] Segars WP , Sturgeon G , Mendonca S , Grimes J , Tsui BM . 4D XCAT phantom for multimodality imaging research. Med Phys. 2010;37:4902‐4915. 20964209 10.1118/1.3480985 PMC2941518

[19] Stelter J , Weiss K , Steinhelfer L , et al. Simultaneous whole‐liver water T1 and T2 mapping with isotropic resolution during free‐breathing. NMR Biomed. 2024;37:e5216. 39099162 10.1002/nbm.5216

[20] Strasser T , Stelter J , Spieker V , et al. Investigating respiratory cycle‐dependent B0 in liver MRI at 3T. Proc Intl Soc Mag Reson Med. 2024;32:4022.

[21] Spicer A , Bawden S , Peggs Z , et al. Characterising the effect of free breathing on abdominal MR spectroscopy and impact on X‐nuclei spectra. Proc Intl Soc Mag Reson Med. 2023;31:3140.

[22] Spieker VJ , Stelter JK , Zimmer VA , et al. Patient‐specific respiratory liver motion analysis for individual motion‐resolved reconstruction. Proc Intl Soc Magn Reson Med. 2023;31:1833.

[23] Rocque M . Fully automated contactless respiration monitoring using a camera. Proceedings of the 2016 IEEE International Conference on Consumer Electronics (ICCE), IEEE; 2016.

[24] Sénégas J , Krueger S , Wirtz D , et al. Comparison of liver motion measured by dynamic MRI and respiration signals obtained by an optical sensor. Proc Intl Soc Mag Reson Med. 2018;26:2528.

