# Supplementary material for: B 0 navigator enables respiratory motion navigation in radial stack‐of‐stars liver Look‐Locker T 1 mapping
Source: Magn Reson Med. 2025 May 20;94(4):1458–68. doi: 10.1002/mrm.30567 (PMC12309899; doi:10.1002/mrm.30567)
Supplement: Supplementary file 1 — Data S1. Supporting Information. [file MRM-94-1458-s001.pdf]

B0 navigator enables respiratory motion navigation in radial stack-of-stars liver T1 mapping

Supplementary information

1 Derivation of the B_0 navigator in a simplified two-species model

The navigator signal for a simplified two-species model, assuming negligible T_2^* decay and uniform coil sensitivities ($c(x, y, z) = 1$) can be expressed as (see Eq. 3 of main manuscript):

$$s_{nav}(T_\mathrm{E}) = \varrho_1 A_1 e^{i2\pi f_{B,1} T_\mathrm{E}} + \varrho_2 A_2 e^{i2\pi f_{B,2} T_\mathrm{E}}. \tag{1}$$

For small field-map-induced phase terms ($f_{B,1}T_\mathrm{E} << 1$ and $f_{B,2}T_\mathrm{E} << 1$), the equation can be approximated using the first-order Taylor expansion $e^x \approx (1 + x)$:

$$\begin{aligned} s_{nav}(T_\mathrm{E}) &\approx \varrho_1 A_1 (1 + i2\pi f_{B,1} T_\mathrm{E}) + \varrho_2 A_2 (1 + i2\pi f_{B,2} T_\mathrm{E}) \\ &= (\varrho_1 A_1 + \varrho_2 A_2) + \varrho_1 A_1 i2\pi f_{B,1} T_\mathrm{E} + \varrho_2 A_2 i2\pi f_{B,2} T_\mathrm{E} \\ &= (\varrho_1 A_1 + \varrho_2 A_2)(1 + i2\pi \frac{\varrho_1 A_1 f_{B,1} + \varrho_2 A_2 f_{B,2}}{\varrho_1 A_1 + \varrho_2 A_2} T_\mathrm{E}) \end{aligned} \tag{2}$$

From this expression, the average field and B_0 navigator estimate can be defined as:

$$f_{B,nav}(\varrho_i, A_i, f_{B,i}) = \frac{\varrho_1 A_1}{\varrho_1 A_1 + \varrho_2 A_2} f_{B,1} + \frac{\varrho_2 A_2}{\varrho_1 A_1 + \varrho_2 A_2} f_{B,2}, \tag{3}$$

Substituting $f_{B,nav}$ back into the expression for $s_{nav}(T_\mathrm{E})$ and reformulating it in exponential form yields:

$$\begin{aligned} s_{nav}(T_\mathrm{E}) &\approx (\varrho_1 A_1 + \varrho_2 A_2)(1 + i2\pi f_{B,nav} T_\mathrm{E}) \\ &\approx (\rho_1 A_1 + \rho_2 A_2) e^{i2\pi f_{B,nav} T_\mathrm{E}} \end{aligned} \tag{4}$$

2 Supplementary Figures

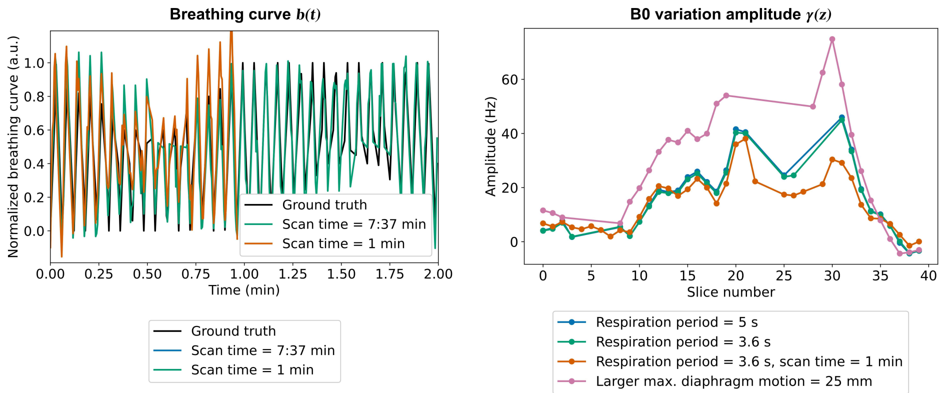

(A) Estimated breathing curve $\boldsymbol{b(t)}$ and B0 variation amplitude $\boldsymbol{\gamma(z)}$

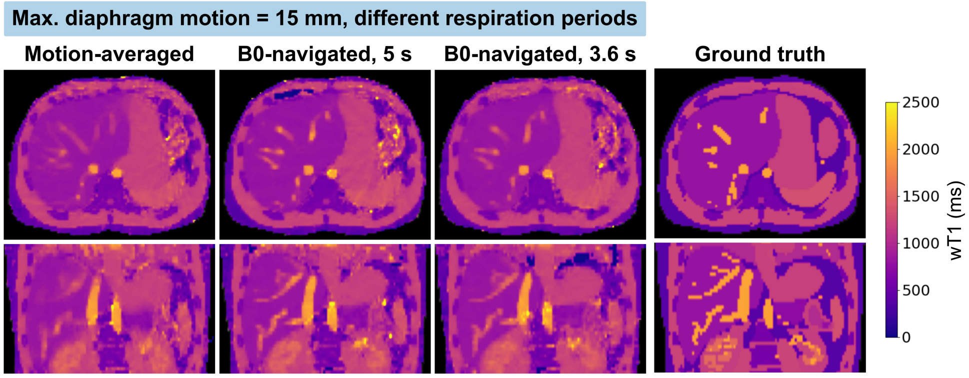

(B) Motion-resolved reconstruction using estimated breathing curve

Figure S1: Simulation with an anatomical body model with a shorter respiration period compared to Fig. 3. The respiration period of 3.6 s is almost synchronized with the 3.5 s interval between inversion pulses, complicating distinguishing between contrast offsets and breathing-induced B_0 variations. (A) The estimated breathing curve aligns with the ground truth even for a shorter scan with a total scan time of 1 min. The estimated B_0 variation amplitude is independent of the respiration period, but shows slight deviations for the shorter 1 min scan. (B) The quality of the B_0-navigated reconstruction is comparable in the simulations of the two different respiration periods.

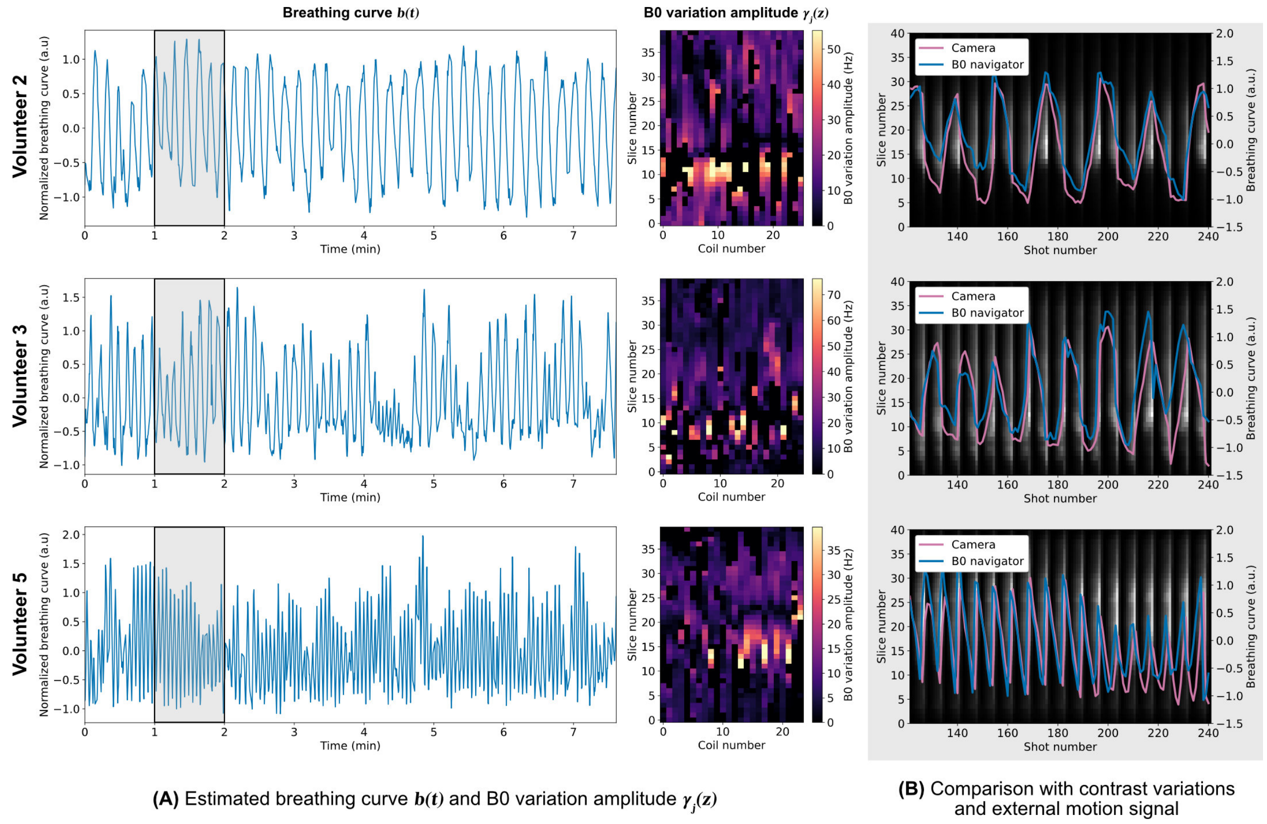

Figure S2: In vivo motion estimation for volunteers 2, 3 and 5. (a) Estimated normalized breathing curve $b(t)$ and B_0 variation amplitude $\gamma_j(z)$ per coil j and slice location z. Note the different scale for the B_0 variation amplitude $\gamma_j(z)$ across the volunteers. (b) The slice-wise magnitude variations from the estimated water navigation signal are shown in a zoomed-in view. The normalized breathing curve and the external motion-tracking camera signal are overlayed in blue and pink, respectively.

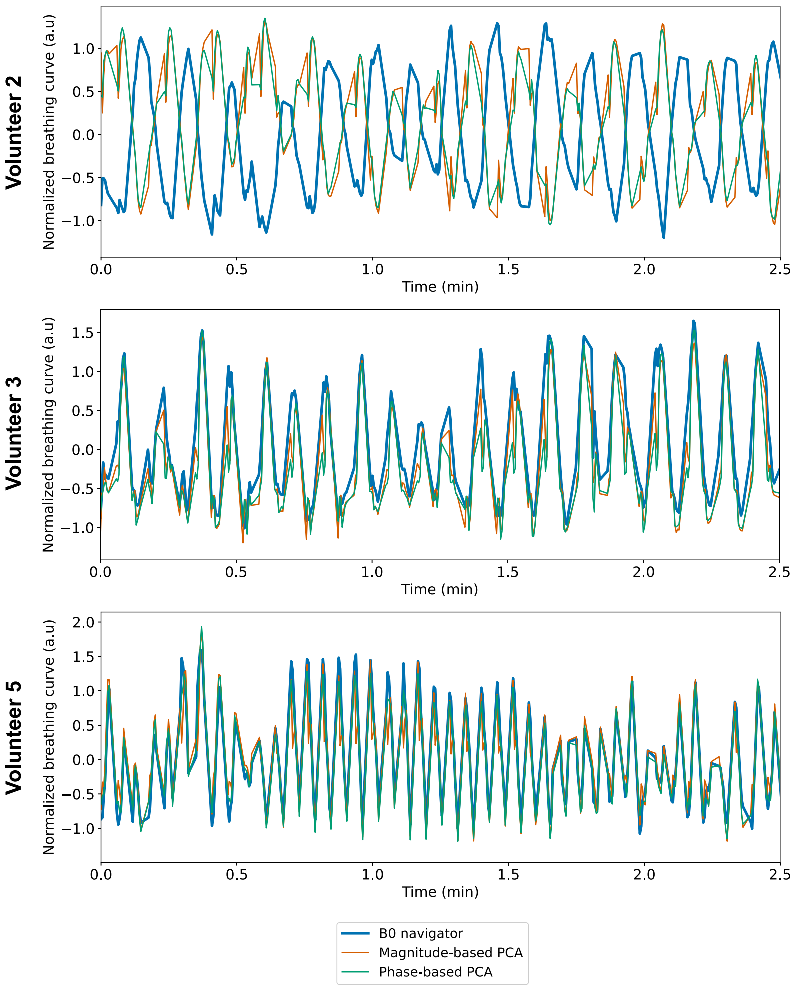

Figure S3: Comparison of the estimated normalized breathing curve $b(t)$ using the B_0 navigator with two different PCA-based motion estimation approaches for volunteers 2, 3 and 5. The magnitude-based PCA estimates motion from the magnitude of the oversampled k-space line, whereas the phase-based PCA estimates motion from the phase of the oversampled k-space line. Both PCA approaches use similar pre-processing with normalization for each T_I contrast. The PCA-based breathing curves are flipped in volunteer 2, while smaller differences in the waveform can be found in volunteers 3 and 5 compared to the B_0 navigator for magnitude- and phase-based PCA.

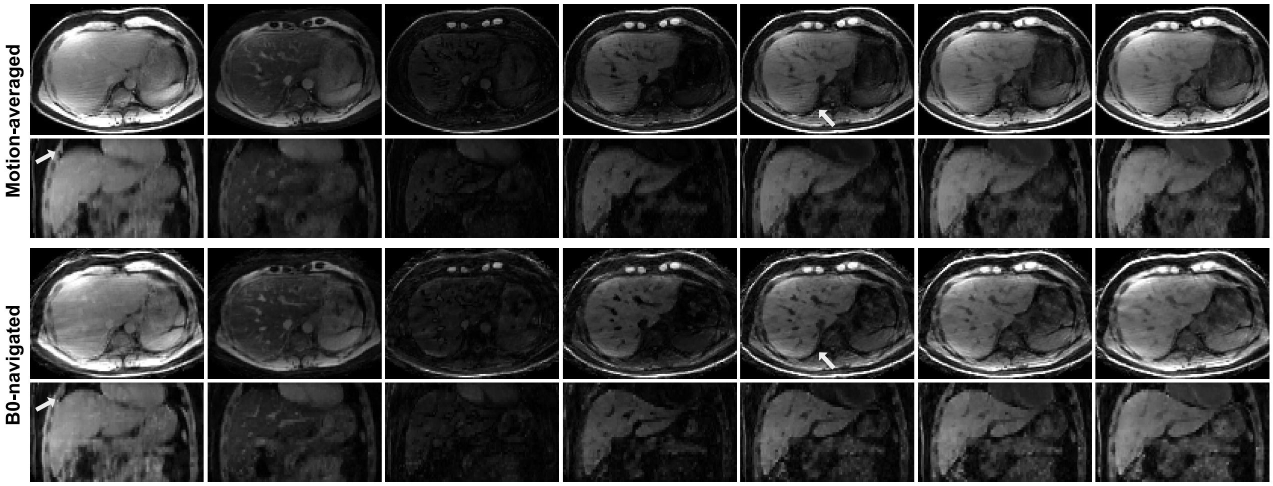

Figure S4: Comparison of the water-separated T_I contrast images for the motion-averaged and B_0-navigated motion-resolved reconstruction. The B_0-navigated reconstruction reduces motion artifacts (white arrows), resulting in considerably less blurring compared to the motion-averaged reconstruction especially in the coronal reformat. The occurrence of undersampling artifacts increases for the motion-resolved reconstruction at the level of the individual T_I contrast images.

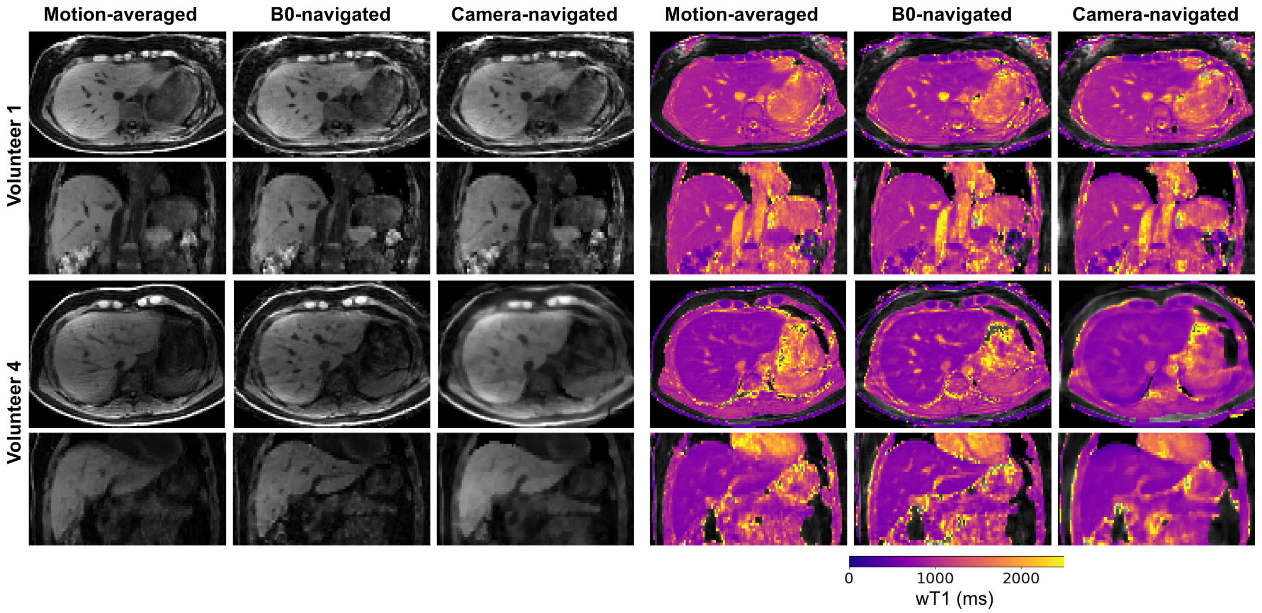

Figure S5: Comparison of motion-resolved reconstruction using the camera as a navigator with motion-averaged and B_0-navigated reconstructions. Water-averaged $T_{I,5}$ images and wT_1 maps are shown for volunteers 1 and 4, demonstrating improved reconstruction quality for the B_0-navigated reconstruction compared to the other methods. The camera-navigated reconstruction appears blurrier, especially in volunteer 4, indicating that the binning of the motion states is not optimal and the reconstructed motion state is severely undersampled. This may be due to a short delay in the camera signal.
